# Subjective and objective outcome in congenital clubfoot; a comparative study of 204 children

**DOI:** 10.1186/1471-2474-8-53

**Published:** 2007-06-28

**Authors:** David Chesney, Simon Barker, Nicola Maffulli

**Affiliations:** 1 Dept of Orthopaedic Surgery, Queen Margaret Hospital, Dunfermline, UK; 2 Dept of Orthopaedic Surgery, Aberdeen Royal Infirmary, Aberdeen, UK; 3 Dept of Orthopaedic Surgery, University of Keele, Staffordshire, UK

## Abstract

**Background:**

Outcome following management of congenital talipes equinovarus (clubfoot) can be assessed in a number of ways. Bjonness stated simply that *"the patient is the final judge of whether he has a good foot*"; a purely subjective assessment. Others have employed objective measures. Combining subjective evaluation with a more objective assessment of movement and position of the foot, is likely to give a more comprehensive picture of the final result of clubfoot. The purpose of this study was to compare subjective and objective outcome following management of clubfoot, and evaluate sex differences in outcome.

**Methods:**

We used a patient-administered subjective assessment of outcome following treatment of clubfoot and compared it with objective anthropometry and range of movement of the ankle to assess and compare subjective and objective outcome in clubfoot. Statistical analysis was performed using Pearson correlation coefficients. Significance was tested using Student's t-test test.

**Results:**

Objective outcome can be assessed using length of the foot, calf circumference and range of movement at the ankle. These are easy to measure, reproducible, and correlate well with subjective outcome. Objective outcome is comparable for boys and girls. However, subjectively, female patients and their parents are less happy with the results of management of clubfoot.

**Conclusion:**

There is a correlation between the anthropometric measures and the subjective outcome and an objective grading can be designed using foot length, calf muscle bulk and range of movement at the ankle.

## Background

Although some authors believe that patients themselves are the ultimate judges of whether they have a 'good foot' [1-4], the need to evaluate outcome of management of clubfoot has traditionally employed objective measures [3]. Combining subjective evaluation of cosmetic acceptability with a more objective assessment of movement and position of the foot is likely to give a more comprehensive picture of the final result of clubfoot.

Objective measures to assess outcome include; range of movement at the ankle [3], leg and foot muscle strength [4], pressure distribution under the sole of the foot [5], calf circumference, gait [6], skinfold thickness, foot length, foot width [7], and shoe size [8]. Radiographic assessment has also been advocated by some, with a number of measurements reported [9], although the use of radiographs is not universally accepted.

As far as we are aware, comparison of objective and subjective measures has, however, not been performed, hence the correlation between the two is unknown. We employed a simple patient-administered subjective assessment compared to an objective assessment using anthropometry and ankle range of movement to compare subjective and objective outcome in clubfoot.

## Methods

Ethics approval for this study was obtained from the Multicentre Ethics Committee, and informed consent was obtained from the parents of each child included in the study.

Two hundred and four children with a diagnosis of idiopathic congenital talipes equinovarus (clubfoot) were identified from the UK talipes study [10]. Subjective assessment was by postal questionnaire, and consisted of three questions assessing satisfaction, function and pain. Questionnaires were sent out addressed to the parent/guardian of the affected child if under 16, or to the child themselves if over 16. The subjective rating therefore reflected the opinion of the patient and the family. The results of the subjective rating were unknown to the interviewer at the time of objective assessment (Table I). Each answer was scored producing a subjective outcome of 15 for a very pleasing result, to 3 for a subjectively poor result.

**Table 1:** Questions posed on subjective by postal questionnaire

Are you satisfied with the outcome of treatment? (please choose one of the following that best describes *what you think*)

□ very satisfied
□ satisfied
□ neither satisfied or dissatisfied
□ dissatisfied
□ very dissatisfied

♦ Does the affected foot function well? (please choose one of the following that best describes *what you think*)

□ there is no limit to daily routine
□ occasionally limited by strenuous exercise
□ usually limited by strenuous exercise
□ occasionally limited by daily routine
□ walking limited

♦ Does the foot ever seem to cause pain? (please choose one of the following that best describes *what you think*)

□ never
□ occasionally on strenuous exercise
□ usually on strenuous exercise
□ occasionally on routine exercise
□ pain on walking

Objective assessment consisted of anthropometric measurements and digital photography, measured during a patient interview by two of the authors (DC & SB). Measurements included maximum calf circumference, foot length and foot width. Calf muscles strength was not used as strength will vary with the age of the child making objective comparison across the group difficult. Skin fold thickness was not measured as this was found to have an unacceptable margin of error when measured on the researchers! Height and weight were also recorded for each child. To assess inter-observer variability, duplicate measures were taken in 14 randomly selected children. There were nine males and five females, of a variety of ages (range 2 years to 28 years), and a mixture of bilateral (8), left sided (2) and right-sided (4) cases.

Two digital photographs were taken for each child. The first was with the child in a crouch position. The photograph was taken just as the heel lifted off the ground (maximal dorsiflexion), the other with the child standing on tiptoes (maximal plantarflexion) [7]. The angles were then measured on computer, and used to calculate range of movement at the ankle. Twelve sets of the photographs were reviewed twice, on separate occasions, to assess repeatability. Ten of the photograph sets were marked by each of us independently to assess interobserver error.

### Statistical analysis

All data was stored using *Microsoft Excel^® ^*and analysed using *SPSS^® ^7.5 for Windows*. All the anthropometric measures followed a normal distribution, as described by Pearson, permitting calculation of correlation coefficients. Significance was tested using Student's t-test. P values are quoted where appropriate throughout the text. We considered p < 0.05 as being statistically significant. Gamma correlation coefficients as described by Goodman and Kruskal were calculated to show reproducibility of the anthropometric measures.

## Results

### Patient epidemiology and management

Two hundred and four children with a diagnosis of idiopathic congenital talipes equinovarus (clubfoot) were identified from the UK talipes study [10]. In each case, the diagnosis was confirmed by the managing consultant orthopaedic surgeon, and management commenced within a few days of birth. Age at the time of study averaged 7 years and 6 months (range 1 year 11 months to 28 years). The male to female ratio was 2.5. 45% children had bilateral clubfoot, 24.3% were left-sided, and 30% right sided disease. All the children were initially managed conservatively. 36% were managed initially with adhesive strapping, 34% with a combination of strapping for the first few weeks of life, and then plaster of Paris casts, and 29% with strapping followed by Denis Browne splints and boots. Surgery was required in 53% (52% of males and 54% of females). In the majority of patients requiring surgery, a posterior or postero-medial release was performed. A small number underwent Achilles tenotomy alone.

### Subjective assessment of outcome

Of the 204 families who took part in the UK Talipes Study, 46 families entered an incomplete response, or no response at all, leaving 158 (77%) complete subjective ratings (Figure 1). Each subjective assessment question response was scored 1–5, five points being given for the answers "very satisfied", "there is no limit to daily routine", and "never" (table 1). Scores therefore ranged from 15 for an excellent overall result, to 3 for a poor overall result.

### Objective assessment

There was a high degree of correlation for all the measures used. (Table 2). To compare children of different ages, height was used as a reference. It was also necessary to have normal anthropometric values to allow assessment of variations in the affected feet measurements. In children with unilateral clubfoot, the unaffected side was used as a normal reference.

**Table 2:** Gamma correlation for inter-observer variation

	Gamma correlation	Standard error	P value
Calf circumference	0.93	0.031	P < 0.001
Foot width	0.77	0.056	P < 0.001
Foot length	0.95	0.024	P < 0.001

In those with bilateral clubfoot, it was necessary to have predicted normal values against which to assess the clubfeet. This was performed using the group of unaffected feet from children with unilateral clubfoot. Sixty-nine children (26 female, 43 male) in the study population had unilateral disease. In this group, there was a very strong linear correlation between foot length and width, and height (Table 3). Calf circumference also displayed a linear relationship with height, but the correlation was less strong, probably because calf circumference is an indirect measure of muscle bulk and will therefore be affected to some degree by lifestyle (Table 3).

**Table 3:** Pearson correlation coefficients for anthropometric measures from the unaffected feet, correlated with height

	Height	Foot length	Foot Width	Calf circumference
Height	1	0.976**	0.857**	0.876**
Foot length		1	0.386**	0.868**
Foot width			1	0.112
Calf circumference				1

n = 69 feet

** significant at the 0.01 level.

Using these data, we calculated normal values for children of a given height, allowing analysis of data from children with bilateral disease.

### Unilateral clubfoot

When the ratio of anthropometric measure for the affected foot compared to the measure for the non-affected foot was calculated and compared to the subjective measure, (table 4), calf circumference ratio was the only measure correlating with the total subjective score. As the ratio decreases (i.e. there is a greater degree of muscle wasting), the outcome was subjectively rated as worse.

**Table 4:** Correlation between objective and subjective measures of outcome for children with unilateral CTEV

	**Satisfaction**	**Function**	**Pain**	**Total**
**Circumference**	0.056	0.289*	0.272*	.271*
**Skin fold**	-0.070	-0.070	0.125	-0.012
**Foot length**	0.289*	-0.040	-0.040	-0.102
**Foot width**	0.201	0.201	-0.052	0.035

n = 65 feet

* correlation significant at 0.05 level.

Circumference also correlated with the function and pain scores, and foot length correlated with satisfaction. There was no evidence of a statistically significant association between foot width and satisfaction (p = 0.097).

### Bilateral clubfoot

To test whether the same correlation existed in children with bilateral clubfoot, it was necessary to use the predicted normal values, calculated using the conversion equations. Ratios of actual-to-expected measures were then calculated, and correlated to the subjective assessment (table 5).

**Table 5:** Correlation between objective and subjective measures of outcome for children with bilateral CTEV

	**Function**	**Pain**	**Satisfaction**	**Total**
**Circumference**	.254*	.260**	-.155	.189
**Fold**	.010	.041	-.050	-.004
**Length**	.050	.124	.256**	.009
**Width**	.059	.226*	-.252**	.061

n = 193 feet

** correlate at .01 level

* correlate at .05 level

While none of the objective measures correlated with the total subjective outcome, calf circumference ratio correlated weakly (p = 0.060). Length and width correlate well with the overall satisfaction. As the foot length discrepancy increases (i.e. the affected foot is shorter), the subjective score is worse.

### All clubfeet

When all the clubfeet were grouped together, using the normal foot for comparison in unilateral cases, and the calculated normal measurements in the bilateral cases, length and width ratios correlated with satisfaction, and calf circumference ratio correlated with function and pain. There was also a good correlation with the overall subjective rating (table 6). Those patients with short, broad feet with extensive muscle wasting had the worst subjective outcome. As the calf circumference ratio increases (i.e. there is less wasting in the clubfoot), the subjective rating increases (Figure 2). Similarly, as the degree of shortening of the affected foot decreases, the satisfaction rating increases (Table 6).

**Table 6:** Correlation between objective and subjective measures of outcome for all children with CTEV

	**Function**	**Pain**	**Satisfaction**	**Total**
**Length**	-.017	.025	-.198*	-.044
**Width**	.109	.125	-.227**	.05
**Circumference**	.256**	.260**	-.091	.210**
**Fold**	.007	.054	-.028	.011

n = 258 feet

** correlate at .01 level

* correlate at .05 level

Using the subjective assessment score, individuals were graded as having excellent, good, or poor results, allowing some quantification of the anthropometric measures for each group. Arbitrarily, children with a subjective score of 15 were considered to have an excellent outcome. Children scoring 13 or 14 were rated as good, and the rest were rated as poor.

Dividing the objective measures into three groups based on the subjective score, mean circumference difference between normal and affected side in patients with unilateral clubfoot was 7% in the poor group, 4% in the good group, and 1% in the excellent group. Because of the small number of children with poor ratings, however, the results were not significant. Nevertheless, there is a trend for the more extreme degree of muscle wasting to be associated with the worse results.

Length ratio correlated with satisfaction score. If these results were grouped into excellent (score 5), good (score 4) and poor (score 1, 2, 3), the difference between good and excellent results was minimal, but children with a poor result on average had a foot length discrepancy of 7%. A short foot correlated with a poor outcome.

### Sex differences in outcome

Subjectively, girls have significantly worse outcomes (t = 2.835, p < 0.005). Objectively, however, comparing males to females, results are not significant for foot length discrepancy (t = 0.775, p = 0.439), or calf circumference (t = 0.109, p = 0.914). Although girls subjectively report a worse outcome, objectively they do not have significantly more muscle wasting or foot shortening.

### Photographic assessment and analysis

Lateral views were taken in 172 feet. Seventeen images did not meet quality control criteria due to compliance problems in the subjects. This included poor views of the ankle and tibia, and variation of greater than 15° from perpendicular to the ankle joint. These images were excluded from further analysis. The remainder were used to measure maximal plantar flexion and maximal dorsiflexion. From these measurements, active range of movement at the ankle was calculated. Both primary researchers (DC & SB) assessed photographs of 10 children. A total of 57 lateral photographs were assessed twice by one investigator. Inter- and intra-observer error, in degrees, are shown in Table 7.

**Table 7:** Inter- and intra-observer variation for ankle range of movement

Interobserver variation	Mean	2.9°
	Standard deviation	2.2°
Intraobserver variation	Mean	3.1°
	Standard deviation	3.1°

Eighty two children who had co-operated with photographic assessment suffered from unilateral clubfoot. The photographs of their normal feet revealed a range of movement from 17.1° to 102.7° (mean 56.4°, SD 19°). The range of movement of the ankles of the affected feet ranged from 0° to 76.9° (mean 33.7° SD 16.6°). Bilaterally affected feet had a range of movement from 0 to 98.6° (mean 36.7° SD 21.5°).

Combining all the clubfoot affected feet, even those feet scoring 15 subjectively have limited ROM compared to normal feet, with a statistically significant correlation between subjective score and ROM (p < 0.05). Ankles with limited range of motion have a worse subjective score.

## Discussion

Outcome following management of clubfoot can be assessed in a number of ways. Bjonness [1] stated simply that *"the patient is the final judge of whether he has a good foot*", with subjective assessments being often used to assess outcome by many authors including [2-5,11]. Subjective assessment is however influenced by a large number of factors, including patient expectations. While one individual may be unhappy at having to attend hospital weekly for plaster cast changes, another may be delighted to receive such dedicated care. Other authors have relied on objective measures; calf muscle strength assessed by gait and toe push-up ability [3], leg and foot muscle strength, measurement of the foot and leg, movement at the ankle and foot, gait analysis [4], muscle strength pressure distribution under the sole of the foot, ankle mobility [5], calf circumference[6], skinfold thickness [7], and shoe size [8].

Radiographic assessment has also been advocated with measurement of Beatson and Pearson's talocalcaneal angle [5,6,9,6,13-15] to evaluate response to different management regimens. The use of radiographs however is not universally accepted. Cummings et al. [16] suggested that radiography was no more reproducible than a subjective assessment, lending strength to the argument that it should be abandoned [5]. Haasbeek and Wright [15] found little relationship between functional and radiographic findings. Indeed, they found it difficult to measure angles on the radiographs because of the overlying tibia and fibula. This became more problematic in older children.

Other authors have found the opposite. Yamamoto's radiographic assessment [17] included lateral and anteroposterior radiographs for measurement of the talocalcaneal angle, the bimalleolo-calcaneal angle (BMC), and the metatarso-talo-bimalleolar angle (MTB). Talocalcaneal angles alone could not be used because of difficulties due to overlying bones. The MTB angle is a measure of the adduction deformity. The BMC angle is a measure of hindfoot varus and talar rotation. Functional results most closely relate to the MTB angle on the anteroposterior radiograph, and the tibiocalcaneal angle on the lateral radiograph. They concluded that these angles were useful as a measure of correction in the immediate postoperative period, and as a method of follow-up.

In the present study, those patients with short, broad feet, calf wasting and stiff ankles had the worst subjective outcome. The length of the affected foot correlates well with satisfaction in patients with unilateral and bilateral clubfoot, but it does not correlate with function or pain, or with the total rating. For patients with unilateral disease, this might be explained in terms of different shoe sizes between feet, and the problems of purchasing footwear. In bilateral disease, however, the feet tend to be similarly affected, with a mean difference in length of only 3.76% (SD 2.38). In this instance, the correlation could be explained by some other factor influencing both foot length and patient satisfaction. Foot width correlates moderately well with satisfaction for all the clubfeet together, and for bilaterally affected children, suggesting that the shape of the foot is important for patient's and parent's satisfaction. Calf circumference correlates well with functional rating, and with the presence of pain. Calf circumference is to some degree, an indirect measure of calf muscle bulk, and it seems likely that the presumed muscle wasting seen in some clubfoot patients causes a reduction in both subjective and objective function of the leg. Diminished calf circumference and muscle wasting is also associated with pain. It seems likely that this may be muscular pain due to fatigue. Calf circumference also correlates with the overall total subjective rating. Range of movement at the ankle is of value in assessing outcome. Although those feet scoring 15 subjectively showed a reduced ROM compared to normal feet, there is, nevertheless, a good correlation between subjective score and ROM, with a correlation coefficient of 0.176 (p < 0.05). Ankles with poor mobility have a worse subjective score.

For the objective assessment of clubfoot, the most useful anthropometric measures were maximal circumference of the calf, and the length of the foot. Range of movement was also confirmed to be a valuable assessment tool.

All the anthropometric measures used are repeatable, with low intra and interobserver errors. Subjective assessment has been criticised for its dependence on patients' personality, expectations and outlook, but a standard against which to compare objective measures is required, and a patient centred subjective assessment such as the three questions used in this study is as valid as other methods reported.

Use of the unaffected foot for comparison in unilateral clubfoot requires that this foot is normal, and it may be more valid to measure normal children to allow calculation of normal values for a given height. Within the confines of the study, however, using the normal foot seems acceptable.

### Does sex affect outcome?

A slightly greater proportion of females required surgery than males [10]. There is a perception among orthopaedic surgeons that girls may have a more form of clubfoot than boys, and therefore have a worse outcome. Our study shows this to be incorrect. Although subjectively, girls had significantly lower scores, objectively there was no significant difference between the sexes. It is likely that parents are less happy with imperfect cosmetic results in girls, and perhaps parental concern regarding cosmesis in part dictates the increased rates of surgery amongst females.

## Conclusion

Outcome following management of clubfoot is subjectively good for the majority of patients, but is influenced by many variables, including perceptions and expectations, and personality, with objectively similar looking feet being subjectively good to some patients and bad to others. Subjective outcome is therefore difficult to use for comparison of different population groups. It should be remembered that the patient's subjective assessment is ultimately all that matters to the patient. We have shown that while subjectively girls have a worse outcome than boys, objectively there is no difference.

Objective outcome can be assessed using length of the foot, calf circumference and range of movement at the ankle. These are easy to measure and are reproducible. There is a correlation between the anthropometric measures and the subjective outcome. An objective grading can be designed using foot length, calf muscle bulk and range of movement at the ankle.

## List of abbreviations

CTEV congenital talipes equinovarus

## Competing interests

The author(s) declare that they have no competing interests.

## Authors' contributions

DC and SB contributed to study design, data collection and analysis, and manuscript preparation. NM contributed to study design, data analysis and manuscript preparation. All authors have read and approved the final manuscript.

## Pre-publication history

The pre-publication history for this paper can be accessed here:


